# A pharmacogenetic study of patients with schizophrenia from West Siberia gets insight into dopaminergic mechanisms of antipsychotic-induced hyperprolactinemia

**DOI:** 10.1186/s12881-019-0773-3

**Published:** 2019-04-09

**Authors:** Diana Z. Osmanova, Maxim B. Freidin, Olga Yu. Fedorenko, Ivan V. Pozhidaev, Anastasiia S. Boiko, Natalia M. Vyalova, Vladimir V. Tiguntsev, Elena G. Kornetova, Anton J. M. Loonen, Arkadiy V. Semke, Bob Wilffert, Nikolay A. Bokhan, Svetlana A. Ivanova

**Affiliations:** 1 Mental Health Research Institute, Tomsk National Research Medical Center of the Russian Academy of Sciences, Aleutskaya str., 4, Tomsk, Russian Federation 634014; 2 National Research Tomsk State University, Lenin Avenue, Tomsk, Russian Federation 36; 3 Department of Twin Research and Genetic Epidemiology, School of Live Course Sciences, King’s College London, Lambeth Palace Road, London, SE1 7EH UK; 4 Research Institute of Medical Genetics, Tomsk National Research Medical Center of the Russian Academy of Sciences, Naberezhnaya Ushaiki str, Tomsk, Russian Federation 10; 5 National Research Tomsk Polytechnic University, Lenin Avenue, Tomsk, Russian Federation 30; 6 Groningen Research Institute of Pharmacy, PharmacoTherapy, Epidemiology & Economics, University of Groningen, Antonius Deusinglaan 1, 9713 AV Groningen, The Netherlands; 7 GGZ Westelijk Noord-Brabant, Hoofdlaan 8, 4661 AA Halsteren, The Netherlands; 8 University Medical Center Groningen, Department of Clinical Pharmacy and Pharmacology, University of Groningen, Hanzeplein 1, 9713 GZ Groningen, The Netherlands

**Keywords:** Dopamine receptors genes, Dopamine transporter *SLC6A3*, Monoamine oxidase (MAO), Antipsychotics, Hyperprolactinemia

## Abstract

**Background:**

Hyperprolactinemia (HPRL) is a classical side effect of antipsychotic drugs primarily attributed to blockade of dopamine D2 receptors (DRD2s) on the membranes of lactotroph cells within the pituitary gland. Certain antipsychotic drugs, e.g. risperidone, are more likely to induce HPRL because of relative accumulation within the adenohypophysis. Nevertheless, due to competition for pituitary DRD2s by high dopamine levels may limit antipsychotic-induced HPRL. Moreover, the activity of prolactin-producing lactotrophs also depends on other hormones which are regulated by the extra-pituitary activity of dopamine receptors, dopamine transporters, enzymes of neurotransmitter metabolism and other factors. Polymorphic variants in the genes coding for these receptors and proteins can have functional significance and influence on the development of hyperprolactinemia.

**Methods:**

A set of 41 SNPs of genes for dopamine receptors *DRD1, DRD2, DRD3, DRD4*, the dopamine transporter *SLC6A3* and dopamine catabolizing enzymes *MAOA* and *MAOB* was investigated in a population of 446 Caucasians (221 males/225 females) with a clinical diagnosis of schizophrenia (according to ICD-10: F20) with and without HPRL who were treated with classical and/or atypical antipsychotic drugs. Additive genetic model was tested and the analysis was carried out in the total group and in subgroup stratified by the use of risperidone/paliperidone.

**Results:**

One statistically significant association between polymorphic variant rs1799836 of *MAOB* gene and HPRL in men was found in the total group. Furthermore, the rs40184 and rs3863145 variants in *SLC6A3* gene appeared to be associated with HPRL in the subgroup of patients using the risperidone/paliperidone, but not with HPRL induced by other antipsychotic drugs.

**Conclusions:**

Our results indicate that genetic variants of *MAOB* and *SLC6A3* may have consequences on the modulation of prolactin secretion. A further search for genetic markers associated with the development of antipsychotic-related hyperprolactinemia in schizophrenic patients is needed.

**Electronic supplementary material:**

The online version of this article (10.1186/s12881-019-0773-3) contains supplementary material, which is available to authorized users.

## Background

Schizophrenia is a serious polymorphic mental disorder, characterized by positive (e.g., hallucinations, delusions), negative (affective flattening, social withdrawal) and cognitive symptoms (disorders of attention, working memory deficits). Treatment of schizophrenic illness usually involves the long-term usage of antipsychotic drugs [1], which have both therapeutic and side effects, related to antagonism to D2 receptors. One of the common side effects of these drugs is hyperprolactinemia [2, 3]. HPRL-related phenomena may lead to additional social stigmatization of the patient with schizophrenia, which reduces the compliance of patients to therapy and increases the cost of treatment. HPRL has short- and long-term consequences that can seriously affect quality of life: menstrual disturbances, galactorrhea, sexual dysfunction, gynecomastia, infertility, and possibly decreased bone mineral density.

Prolactin (PRL) is a polypeptide hormone that contains 199 amino acids and is categorized as part of the growth hormone family [4]. Prolactin secretion is under a complex neuroendocrine control, in which various agents participate: neurotransmitters, biologically active neuropeptides, hormones of peripheral endocrine glands. Synthesis and secretion of prolactin are carried out by lactotrophs of the pituitary gland, which constitute an average of 20–50% of the total cell population of the pituitary cells [5].

Factors involved in the regulation of prolactin secretion can be divided into two groups: 1. prolactin-inhibiting – dopamine (DA), gamma-aminobutyric acid, gastrin, somatostatin, gonadotropin-binding protein and 2. prolactin-stimulating – serotonin, thyrotropin-releasing hormone (TRH), gonadotropin-releasing hormone (TRG), vaso-intestinal peptide, opiates, neurotensin and substance P, oxytocin, angiotensin 2 [6, 7].

Dopamine holds a predominant role in the regulation of prolactin secretion. Dopamine, which is released by tuberoinfundibular neurons, normally acts at specific receptors on lactotrophes to tonically inhibit prolactin secretion and maintain physiologically normal prolactin levels in the blood [8]. These dopamine receptors located on lactotroph membranes belong to the D2 subclass of the dopamine receptor family [9, 10].

The hypothalamic dopaminergic neurons, which provide DA to the anterior pituitary gland, are themselves regulated by feedback from PRL through a ‘short-loop feedback mechanism’. Elevation of serum levels of prolactin increases hypothalamic dopamine synthesis [11] and the concentration of dopamine in hypothalamic-pituitary portal blood [12].

All typical antipsychotic medications are associated with sustained hyperprolactinemia due to their high affinity for the D2 receptor and their slow dissociation from the receptor once bound, but the atypicals clozapine and quetiapine differ quite dramatically in their propensity to cause prolonged high prolactin levels, because they are loosely bound to D2 receptors [13]. The dopamine D2 partial receptor agonist aripiprazole is even capable to reduce prolactin levels [3].

Another way to explain hyperprolactinemia is the ability of antipsychotic-drugs to cross the blood-brain barrier and to affect the activity of prolactin-inhibiting and prolactin-stimulating factors. This capacity partly depends upon affinity to the drug efflux transporter P-glycoprotein, which prevents drugs to pass the blood brain barrier [14, 15]. This may result in relatively accumulation within the pituitary gland in comparison to brain tissue which is protected by the blood brain barrier [16].

PRL elevations with antipsychotic medication generally are dose dependent. However, antipsychotics having a high potential for PRL elevation (amisulpride, sulpriride, risperidone and paliperidone) can have a profound impact on PRL levels even at relatively low doses, while PRL levels with antipsychotics having a minimal effect on PRL, in most cases, can remain unchanged (quetiapine) or reduce (aripiprazole) over all dosages. Although tolerance and decreases in PRL values after long-term administration of PRL-elevating antipsychotics can occur, the elevations, in most cases, remain above the upper limit of normal [3].

In conclusion, antipsychotic drugs may affect prolactin release directly by blocking dopamine D2 receptors on lactotrophs, but modulate this activity by affecting dopamine levels within the hypothalamic-pituitary portal blood. Moreover, antipsychotics can modulate intracerebral mechanisms involved in the regulation of prolactin release. As the main targets for the therapeutic actions of neuroleptics in schizophrenia are components of cerebral dopamine and – to a lesser extent – serotonin neurotransmission, the authors aimed at identifying the association of neuroleptic hyperprolactinemia with polymorphism of genes that determine the activity of various components of dopaminergic transmission – enzymes that catalyze the synthesis and metabolism of dopamine, its storage, release into the synaptic cleft and reverse capture, as well as the structure of its receptors.

According to the literature data genetic variants of dopaminergic receptors, transporters and enzyme may have functional consequences on the respond of antipsychotic treatment and development of side effects of therapy.

In the structure of the dopamine receptor D1 (localized on the postsynaptic membrane), two single nucleotide polymorphisms associated with the risk of developing schizophrenia have been identified: *G198A* and *G1263A* [17]. These substitutions are synonymous, they do not lead to a change in the amino acid composition of the receptor, but presumably may have an effect on gene expression, particularly on the formation of heterodimeric forms of the dopamine receptor consisting of the D1 and D2 subunits [18]. In several studies rs4532 polymorphism in *DRD1* is considered as a potential pharmacogenomic marker for treatment response to antipsychotic drugs [19, 20].

At least 23 single nucleotide polymorphisms within *DRD2* were evaluated to determine whether any of them controlled *DRD2* expression [21]. The polymorphism of D2 receptors *Taq1A*, represented by two alleles – *A1* and *A2* in the region of the promoter that regulates gene expression, has been studied most. It was found that reduced dopamine D2 receptor binding in the human striatum associated with the A1 allele [22]. The association of *Taq1A* polymorphism with schizophrenia was reported [23], it was later shown that *A1* allele is associated with a predisposition to the development of dyskinesias on the background treatment with neuroleptics [24]. The study of this polymorphism is complicated by the fact that this site is “concurrently” promoted by another, located next to the gene – *ANNK1*, which encodes protein kinase *RKK2*. DRD2/ANKK1 genotypes are associated with susceptibility to second generation antipsychotic-induced akathisia [25].

Polymorphism of other subtypes of dopamine receptors of the second type, D3 and D4, also, according to a number of researchers, is associated with a risk of developing schizophrenia spectrum disorders [26–28]. There is evidence that the several polymorphic variants in gene D3 receptor are associated with a risk of developing schizophrenia [29], as well as with a decrease in the level of implementation cognitive tasks [30] and antipsychotic induced tardive dyskinesia [31].

The dopamine transporter gene (*SLC6A3*) appears to be one of the most important candidate genes for affecting mental disorders, as it is the target of many psychostimulants that cause symptoms similar to the positive symptoms of schizophrenia. Controversial information was obtained by studying the effect of polymorphism in *SLC6A3* on pharmacogenetics of adverse events in schizophrenia treatment [32, 33].

Dopamine can be degraded in several ways, in which enzymes such as monoamine oxidase (MAO) and catechol-O-methyltransferase (COMT) play a leading role. There are two isoforms of monoamine oxidase – MAOA and MAOB, whose genes are located on the X chromosome. MAOA catalyzes monoamine neurotransmitters including 5-hydroxytryptamine (5-HT, serotonin), norepinephrine and dopamine, while MAOB deaminates 2-phenylethylamine and also dopamine [34]. The association of polymorphic variants of the *MAOA* gene with features of an alarming series, such as neuroticism and negative affect, is described. Changes in the sequence of nucleotides in the promoter region of the *MAOA* gene are associated with a reduced level of expression of this gene and may be associated with an increased level of aggression and impulsivity compared to a typical *MAOA* promoter that determines the normal level of expression [35].

There is a series of studies with the search for associations of polymorphisms of the genes of dopamine receptors and metabolizing enzymes in various combinations and response or side effects to antipsychotic therapy: *DRD2* and dopamine transporter *SLC6A3* [36]; *DRD1, DRD2, DRD3* and *COMT* [37]; *DRD1, COMT, DRD2,* and *DRD3* [38].

## Methods

Based upon reviewing the literature we selected a set of 26 polymorphisms in dopamine receptors genes (*DRD1, DRD2, DRD3, DRD4*), 12 polymorphic variants of dopamine transporter *SLC6A3*, as well as 3 polymorphisms of monoamine oxidase A and B and here we present new data on the association between them and HPRL in antipsychotic drug-treated patients with schizophrenia from West Siberia, Russian Federation.

### Patients

The study population was previous described by Ivanova et al. [39, 40]. The work was carried out in accordance with The Code of Ethics of the World Medical Association (Declaration of Helsinki 1975, revised in Fortaleza, Brazil, 2013) for experiments involving humans. The patients in this study were retrieved from three psychiatric hospitals in Tomsk, Kemerovo, and Chita oblasts in Siberia. The inclusion criteria were a clinical diagnosis of schizophrenia according to ICD-10 (F20), patients aged 18–75 years, Caucasian physical appearance and a signed informed consent form to participate in the study after approval from the study (protocol N63/7.2014) from the Local Bioethics Committee of the Mental Health Research Institute. Exclusion criteria for all patients were non-Caucasian physical appearance (e.g., Mongoloid, Buryats or Khakassians), pregnancy or relevant gynecological and endocrine (thyroid) disorders, relevant pharmacological withdrawal symptoms or organic brain disorders (e.g., epilepsy, Parkinson’s disease), the presence of acute and chronic infectious, inflammatory, autoimmune diseases, as well as persons with active oncological diseases, accompanied by an increased level of prolactin on the blood.

The total sample consisted of 446 patients (221 males/225 females). The women were significantly older (*p* = 2.6e-8, Mann-Whitney test (MWT)) than the men (mean ± SD 45.2 ± 13.9 vs. 37.8 ± 11.9 years). Among the women, 86 were > 50 years of age. Women suffered from the disease for a significantly longer (*p* = 0.0002, MWT) period of time (17.6 ± 12.5 vs. 13.1 ± 10.0 years). The median antipsychotic daily dose was 500 mg CPZeq (quartiles 280; 750) in men and 320 mg CPZeq (quartiles 200; 750) in women (p = 0.0002, MWT).

A total of 227 patients suffered from HPRL (98 males/129 females) according to the predefined criteria [3, 39]. Demographic and clinical features of patients with schizophrenia with and without hyperprolactinemia are presented in Table 1.

**Table 1:** Demographic and clinical features of patients with schizophrenia with and without HPRL

Feature	Patients with HPRL, *n* = 227	Patients without HPRL, *n* = 219	*p*-value
Age	49.19 ± 13.19	42.94 ± 13.56	0.031
Male/Female	98/129	123/96	0.006
Dose of antipsychotics into chlorpromazine equivalent	400 (225; 750)	400 (280; 750)	0.074
Duration of the disease	11.0 (4.0; 22.0)	14.0 (8.0; 22.0)	0.041

One hundred ninety-one patients were treated with conventional antipsychotics in oral and/or long-acting formulations. The most common conventional antipsychotic was haloperidol, which was used in 110 patients, but other treatments included oral chlorpromazine, chlorprothixene, trifluoperazin, and zuclopenthixol, and/or long-acting formulations of haloperidol-, zuclopenthixol-, and flupenthixol-decanoate. A total of 176 patients were treated with atypical antipsychotics: risperidone, clozapine, quetiapine, olanzapine, amisulpride, paliperidone, and sertindole. Different combinations of classical and atypical drugs were used by 79 patients. 76 patients were treated with risperidone/paliperidone.

To compare antipsychotic medications, all dosages were converted into chlorpromazine equivalents (CPZeq) [41].

Blood samples were taken 8 h after overnight fasting in tubes containing EDTA for DNA extraction and in tubes with CAT (clot activator) to obtain serum (BD Vacutainer). Blood with EDTA was stored in several aliquots at -20 °C until DNA isolation. Blood samples with CAT were centrifuged for 30 min at 1500 rpm at 4 °C to obtain serum.

### Hormone analysis

The PRL concentration was measured in serum using the AccuBind ELISA Microwells kit (Monobind Inc., USA). In this microplate immunoenzymatic assay, the ELISA has a sensitivity of 0.004 ng/well. This is equivalent to a sample containing 0.150 ng/ml PRL. The upper limits for normal PRL concentration were set at ≤20 ng/ml for men and ≤ 25 ng/ml for non-pregnant, non-nursing women [39, 40]. For women of reproductive age, blood was when possibly taken in the follicular phase of the menstrual cycle. This corresponds to the criteria for HPRL applied by Kelly et al. [42] and Peuskens et al. [3].

### DNA analysis

DNA was isolated from the leukocytes in whole peripheral blood from patients with mental disorders using the standard phenol-chloroform method.

Genotyping was carried out for *DRD1, DRD2, DRD3, DRD4,* and *SLC6A3* genes in the Genome Analysis Facility, Dept. Genetics (Head: Prof. Dr. C. Wijmenga), University Medical Center Groningen on the MassARRAY® Analyzer 4 (Agena Bioscience™) using the set SEQUENOM Consumables iPLEX Gold 384. DNA sample preparation for SEQUENOM MassARRAY® Analyzer 4 includes several steps: a standard PCR reaction to obtain the amplification products, a shrimp alkaline phosphatase (SAP) reaction to neutralize the unincorporated dNTPs in the amplification products, the PCR iPLEX Gold extension reaction, and then placing the samples on a special chip (SpectroCHIP Array) using NanoDispenser RS1000 prior to loading them into the analyzer. SNPs in *MAOA*, *MAOB* genes were genotyped in the Mental Health Research Institute, Tomsk National Research Medical Center of the Russian Academy of Sciences, using the fluorogenic 5′-exonuclease TaqMan technology performed on the real-time polymerase chain reaction system “StepOnePlus” (Applied Biosystems, USA).

We selected a set of 26 SNPs from the following dopamine receptors genes: *DRD1, DRD2, DRD3, DRD4* (Table 2), 12 polymorphic variants of dopamine transporter *SLC6A3* and 3 polymorphisms of genes *MAOA* and *MAOB* (Table 3).

**Table 2:** List of analyzed polymorphic variants of dopamine receptor genes

Gene	SNP	Chromosome	Chromosome position	Alleles	Minor Allele Frequency
*DRD1*	rs4532	5	174,870,150	T/C	24.4
*DRD2*	rs6275	11	113,283,477	C/T	47.3
*DRD2*	rs6277	11	113,283,459	C/T	24.4
*DRD2*	rs1076560	11	113,283,688	C/A	22.9
*DRD2*	rs1801028	11	113,283,484	C/G	3.1
*DRD2*	rs4245147	11	113,318,007	T/C	45.4
*DRD2*	rs2283265	11	113,285,536	G/T	22.7
*DRD2*	rs6279	11	113,281,073	G/C	47.8
*DRD2*	rs1076562	11	113,296,008	G/A	42.1
*DRD2*	rs2734842	11	113,280,274	G/C	46.7
*DRD2/ANKK1*	rs2734849	11	113,270,160	T/C	24.5
*DRD3*	rs11721264	3	113,879,404	G/A	41.4
*DRD3*	rs167770	3	113,879,562	A/G	42.2
*DRD3*	rs3773678	3	113,870,078	C/T	32.0
*DRD3*	rs963468	3	113,862,887	G/A	27.2
*DRD3*	rs7633291	3	113,887,068	T/G	22.4
*DRD3*	rs2134655	3	113,858,201	G/A	20.3
*DRD3*	rs9817063	3	113,847,108	C/T	42.8
*DRD3*	rs324035	3	113,868,854	C/A	40.2
*DRD3*	rs1800828	3	113,891,549	G/C	22.2
*DRD3*	rs167771	3	113,876,275	A/G	41.1
*DRD3*	rs6280	3	113,890,815	T/C	48.6
*DRD3*	rs1587756	3	113,902,751	T/C	14.6
*DRD4*	rs3758653	11	636,399	T/C	24.7
*DRD4*	rs11246226	11	641,191	A/C	47.7
*DRD4*	rs936461	11	636,496	G/A	45.7

**Table 3:** List of analyzed polymorphic variants of the dopamine transporter gene and the monoamine oxidase genes

Gene	SNP	Chromosome	Chromosome position	Alleles	Minor Allele Frequency
*SLC6A3*	rs3756450	5	1,448,148	T/C	34.9
*SLC6A3*	rs2550956	5	1,447,841	T/C	17.3
*SLC6A3*	rs6347	5	1,411,412	A/G	29.8
*SLC6A3*	rs2617605	5	1,442,521	A/G	25.6
*SLC6A3*	rs3863145	5	1,392,711	C/T	16.8
*SLC6A3*	rs250686	5	1,425,159	G/A	40.7
*SLC6A3*	rs464049	5	1,423,905	C/T	39.1
*SLC6A3*	rs4975646	5	1,433,401	G/A	13.4
*SLC6A3*	rs1048953	5	1,438,174	C/T	14.6
*SLC6A3*	rs11133767	5	1,401,580	A/G	31.8
*SLC6A3*	rs27048	5	1,412,645	C/T	32.1
*SLC6A3*	rs40184	5	1,395,077	G/A	41.3
*МАОА*	rs6323	X	43,591,036	G/T	37.5
*МАОА*	rs1137070	X	43,603,391	C/T	44.8
*МАОB*	rs1799836	X	43,627,999	A/G	45.6

### Statistical analysis

We analyzed associations between the polymorphisms and HPRL using logistic regression models including HPRL as a dependent categorical variable and polymorphisms as the predictors. Age, sex, and CPZeq were used as covariates. Additive genetic model was tested and Odds ratios (OR) along with 95% confidence intervals (CI) were calculated regarding the risk of HPRL for the rare allele vs common allele. Prior to the analysis, the polymorphisms were filtered out in case of minor allele frequency below 5% or Hardy-Weinberg equilibrium (HWE) *p*-value below 0.001. HWE was tested using Fisher’s exact test except for SNPs located in the X-chromosomal (*MAOA*, *MAOB*). The Mann-Whitney test was used to compare qualitative traits and χ^2^ test for categorical traits.

All calculations were performed in the R statistical environment using basic R functions and the SNPassoc package [43].

## Results

From the list of 38 SNPs studied of dopamine receptor genes and *SLC6A3* genes we excluded 4 SNPs (rs6275, rs6347, rs2550956, rs11133767) with Hardy-Weinberg equilibrium *p*-value < 0.001, thus leaving 34 SNPs for the analysis of associations with HPRL. In the total group of patients none of the studied genetic markers localized in autosomal chromosomes are associated with HPRL (Additional file 1).

Polymorphic variants of monoamine oxidase genes are localized in the X chromosome. Taking into account the hemizygotic status of X-chromosomal markers for men, the analysis of these markers was carried out separately in men and women. Using the logistic regression analysis with age and CPZeq as covariate we found the association of rs1799836 with HPRL in men (Table 4). Allele and genotype frequencies are shown in Additional file 2. A protective effect of the rs1799836*A allele against HPRL development was observed.

**Table 4:** Analysis of association between HPRL and the polymorphisms of *MAO* genes for all patients divided by gender

Gender	SNP	OR	95% CI Lower bound	95% CI Upper bound	*p*-value
Females	rs1799836	0.939	0.644	1.369	0.744
	rs1137070	0.757	0.497	1.153	0.195
	rs6323	0.786	0.512	1.208	0.273
Males	rs1799836	0.748	0.561	0.998	0.048*
	rs1137070	1.279	0.942	1.737	0.115
	rs6323	1.315	0.970	1.782	0.078

Logistic regression analysis was used to test additive genetic model for association between HPRL and the *MAO* genes polymorphisms in males and females separately adjusting for age and CPZeq. OR – odds ratio; CI – lower and upper bound 95% confidence intervals. ORs are reported for the risk of HPRL attributable to the rare allele vs common allele

*Values are deemed to have significance

Due to the fact that according to literature data, HPRL is significantly more common in patients receiving risperidone/paliperidone, the next step was the analysis in the subgroup of patients using the risperidone/paliperidone. Using logistic regression with age, gender, and CPZeq as covariates we found that the rs40184 and rs3863145 variants in *SLC6A3* gene appeared to be associated with HPRL in this subgroup of patients (Table 5). Allele and genotype frequencies are shown in Additional file 3. The rs40184*A and the rs3863145*C alleles were found to exhibit a protective effect, reducing the risk of hyperprolactinemia in carriers of these genotypes. Notably, the both SNPs are in some linkage disequilibrium LD, therefore, their association with the HPRL is bound by the same haplotype (Additional file 4). Polymorphisms in *MAO* genes were not associated with hyperprolactinemia in the studied subgroup (Additional file 5).

**Table 5:** Analysis of association between HPRL and the genetic polymorphisms in patients from risperidone/paliperidone group

Gene	SNP	OR	95% CI Lower bound	95% CI Upper bound	*p*-value
*DRD1*	rs4532	0.522	0.216	1.261	0.148
*DRD2*	rs1076562	0.490	0.192	1.249	0.135
*DRD2*	rs4245147	1.148	0.519	2.541	0.733
*DRD2*	rs2283265	0.900	0.301	2.686	0.850
*DRD2*	rs2734842	0.472	0.187	1.194	0.113
*DRD2*	rs6277	2.081	0.864	5.011	0.102
*DRD2*	rs6279	0.434	0.164	1.145	0.092
*DRD2*	rs1076560	0.969	0.327	2.878	0.955
*DRD2/ANKK1*	rs2734849	1.833	0.781	4.302	0.164
*DRD3*	rs11721264	2.042	0.677	6.156	0.205
*DRD3*	rs2134655	0.659	0.203	2.140	0.488
*DRD3*	rs963468	0.647	0.251	1.666	0.367
*DRD3*	rs167771	1.150	0.244	5.414	0.859
*DRD3*	rs324035	1.719	0.380	7.783	0.482
*DRD3*	rs167770	1.963	0.622	6.195	0.250
*DRD3*	rs7633291	2.462	0.640	9.473	0.190
*DRD3*	rs9817063	1.635	0.626	4.270	0.316
*DRD3*	rs1587756	2.407	0.489	11.861	0.280
*DRD3*	rs1800828	2.813	0.739	10.711	0.129
*DRD3*	rs3773678	1.556	0.326	7.412	0.579
*DRD3*	rs6280	2.153	0.668	6.947	0.199
*DRD4*	rs3758653	1.403	0.410	4.806	0.590
*DRD4*	rs936461	1.500	0.577	3.895	0.405
*DRD4*	rs11246226	2.421	0.955	6.142	0.063
*SLC6A3*	rs27048	1.419	0.660	3.054	0.370
*SLC6A3*	rs3756450	1.373	0.512	3.684	0.529
*SLC6A3*	rs40184	0.341	0.137	0.852	0.021*
*SLC6A3*	rs4975646	0.640	0.258	1.589	0.336
*SLC6A3*	rs2617605	0.880	0.337	2.299	0.794
*SLC6A3*	rs464049	0.538	0.209	1.384	0.199
*SLC6A3*	rs1048953	0.823	0.337	2.008	0.668
*SLC6A3*	rs250686	0.563	0.214	1.482	0.245
*SLC6A3*	rs3863145	0.362	0.135	0.970	0.043*

Logistic regression analysis was used to test an additive genetic model for association between HPRL and the polymorphisms in adjusting for age, sex and CPZeq. OR – odds ratio; CI – lower and upper bound 95% confidence intervals. ORs are reported for the risk of HPRL attributable to the rare allele vs common allele

*Values are deemed to have significance

## Discussion

We studied the association between polymorphisms of genes relevant for dopaminergic neurotransmission such as receptors, transporter, and enzymes with antipsychotic drug-induced HPRL in white patients with schizophrenia from Siberia. We excluded patients with physiological or pathological conditions that may affect PRL secretion and corrected for variables related to PRL secretion and/or that may determine antipsychotic drug load. The assessment of a possible association is considered the first step in discovering the possible functional consequences of genetic variations. We distinguished HPRL for men and women. The studied women were significantly older than the men and patients with HPRL were more often female than those without. Premenopausal women have significantly higher PRL levels than postmenopausal female persons, who have not significantly different levels in comparison to men [44–46]. This might be related to modulation of PRL dynamics by estrogen levels [44]. The gender and age differences between the patients with and without HPRL may have falsely decreased the actual prevalence of drug-induced HPRL in the HPRL group in comparison to non-HPRL patients. However, when only male patients were studied significant association was found with one polymorphism rs1799836 of *MAOB* gene.

*MAOB*, located adjacent to *MAOA* on the opposite strand at chromosome Xp11.23, is involved in the breakdown of dopamine in the brain [34]. MAO-B is widely distributed within the brain with moderate levels within the adenohypophysis – where MAO-A is low – and hypothalamus [47, 48]. A non-coding single nucleotide polymorphism rs1799836 in intron 13 is associated with Parkinson’s disease [49, 50], schizophrenia [51] and is also significantly associated with reduced negative emotionality [52]. Further, a case-control study by Gasso et al. indicates that the G allele is a risk factor for developing schizophrenia in a Spanish population [53].

Kang S.G. et al., reported that males schizophrenic patients with *MAOA* 3-repeat uVNTR and *MAOB* A644 genotype has higher association with antipsychotic-induced restless leg syndrome [54].

A. C. Need et al. (2006) studied whether obesity is associated with genetic variants that increase the availability of dopamine. Low activity genotypes at both the *MAOA* and *MAOB* loci – showed a relative risk for obesity of 5.01 [55]. Antipsychotic-induced weight gain, including as a consequence of increasing the level of prolactin, can be a particular problem of in schizophrenia treatment, often causing non-compliance and consequent relapse. It is possible that functional variants in the *MAOA* and *MAOB* genes could predispose to antipsychotic-induced weight gain in patients with schizophrenia.

Our preliminary hypothesis was that genetic variants of dopamine receptors may have functional consequences on the modulation of PRL secretion and may play an important role in the development of hyperprolactinemia, but the results of the study indicate that there is no association in the group of patients.

However we found that rs40184 and rs3863145 of *SLC6A3* gene are associated with risperidone/paliperidone induced HPRL, but not with HPRL induced by other antipsychotic drugs. The solute carrier family 6 (neurotransmitter transporter), member 3 (*SLC6A3*) gene encodes the dopamine transporter DAT. The difference between risperidone/paliperidone and other antipsychotics might indicate that, although dopamine transporters are present within the adenohypophysis [56] and may have a role in prolactin secretion [57], the pharmacological mechanism related to this association is less likely on the level of the pituitary gland (which is outside the blood-brain-barrier) but localized within the brain itself. Risperidon/paliperidone relatively accumulates within the pituitary gland in comparison to other antipsychotics [16] and thus can be expected to bind to a higher extend to dopamine D2 receptors there.

The DAT protein is expressed in the membrane of neurons, where it transports dopamine from the synaptic cleft back into neurons for reuse. *SLC6A3* plays a critical role in controlling dopamine transmission (spatial and temporal domains) through the accumulation of dopamine in extracellular space which is major site of action of psychostimulant drugs [58–60]. The results of several studies indicate that altered *SLC6A3* functioning may lead to inter-individual variability of response to antipsychotic drug treatment. Sjoholm H. et al*.* (2004) [61] reported increased number of *SLC6A3* binding sites in the schizophrenia patients who were being treated with dopamine D2-receptor blocking antipsychotics. Single locus analysis showed significant association of nine variants from *SLC6A3*, *PIP4K2A* and *BDNF* genes with incomplete antipsychotic response in schizophrenia patients with high severity [62]. The multifactor-dimensionality reduction approach identified gene-gene interaction among *BDNF*_rs7103411-*BDNF*_rs1491851-*SLC6A3*_rs40184 in severely ill incomplete responders [62]. Hence, at least some intracerebral pharmacological effects of altered *SCL6A3* functioning can be expected and these may also influence PRL secretion.

Our new data could be taken into account when therapy with risperidone/paliperidone or different antipsychotic therapy is considered regarding the risk of developing HPRL.

## Conclusions

In conclusion, we found an association of antipsychotic-induced HPRL with rs1799836 of the *MAOB* gene in (hemizygous) men and rs40184 and rs3863145 variants in the *SLC6A3* gene which codes for the dopamine transporter in patients using risperidone/paliperidone. Strength of our study is the relatively large patient population and careful assessment of hyperprolactinemia. A relative weakness is the sex difference between the HPRL versus non-HPRL groups and the relatively high age of women as this may increase the number of post-menopausal women in the HPRL-group. However, this also makes the associations which we found more likely to exist. A further search for genetic markers associated with the development of antipsychotic-related hyperprolactinemia in schizophrenic patients is needed.

## Additional files

Additional file 1: **Table S1.** Logistic regression analysis for HPRL as a dependent categorical variable and polymorphisms as the predictors and age, sex, CPZeq as covariates for all patients. (DOC 65 kb)

Additional file 2: **Table S2.** Genotype and allele frequencies for *MAO* gene polymorphisms. (DOC 45 kb)

Additional file 3: **Table S3.** Genotype and allele frequencies for all polymorphisms of studied genes in the subgroup of patients using the risperidone/paliperidone. (DOC 275 kb)

Additional file 4: **Table S4.** Lewontin’s D’ statistics to measure pairwise linkage disequilibrium between *SLC6A3* SNPs. (XLS 40 kb)

Additional file 5: **Table S5.** Analysis of association between HPRL and polymorphisms in *MAO* genes for female/male patients in risperidone/paliperidone group. (DOC 34 kb)

## Abbreviations

CI: Lower and upper bound 95% confidence intervals
COMT: Catechol-O-methyltransferase
CPZeq: Chlorpromazine equivalents
DA: Dopamine
DRD2s: Dopamine receptor D2
HPRL: Hyperprolactinemia
HWE: Hardy-Weinberg equilibrium
ICD-10: International Statistical Classification of Diseases and Related Health Problems 10th Revision
MAO: Monoamine oxidase
MWT: Mann-Whitney test
OR: Odds ratio
PRL: Prolactin
SNP: Single nucleotide polymorphism
TRG: Gonadotropin-releasing hormone
TRH: Thyrotropin-releasing hormone
