# Supplementary material for: A pharmacogenetic study of patients with schizophrenia from West Siberia gets insight into dopaminergic mechanisms of antipsychotic-induced hyperprolactinemia
Source: BMC Med Genet. 2019 Apr 9;20(Suppl 1):47. doi: 10.1186/s12881-019-0773-3 (PMC6454588; doi:10.1186/s12881-019-0773-3)
Supplement: Supplementary file 1 — Table S1. Logistic regression analysis for HPRL as a dependent categorical variable and polymorphisms as the predictors and age, sex, CPZeq as covariates for all patients. (DOC 65 kb) [file 12881_2019_773_MOESM1_ESM.doc]

**Supplementary table 1**

Logistic regression analysis for HPRL as a dependent categorical variable and polymorphisms as the predictors and age, sex, CPZeq as covariates for all patients

| Gene | SNP | OR | 95% CI  Lower bound | 95% CI  Upper bound | *p*-value |
| --- | --- | --- | --- | --- | --- |
| *DRD2* | rs1076562 | 0.859 | 0.639 | 1.156 | 0.316 |
| *DRD3* | rs11721264 | 1.000 | 0.718 | 1.394 | 0.999 |
| *DRD3* | rs2134655 | 1.065 | 0.774 | 1.464 | 0.700 |
| *SLC6A3* | rs27048 | 1.065 | 0.817 | 1.387 | 0.642 |
| *SLC6A3* | rs3756450 | 1.004 | 0.717 | 1.406 | 0.981 |
| *DRD4* | rs3758653 | 1.088 | 0.740 | 1.598 | 0.668 |
| *SLC6A3* | rs40184 | 1.088 | 0.833 | 1.422 | 0.534 |
| *DRD2* | rs4245147 | 0.959 | 0.734 | 1.252 | 0.759 |
| *DRD1* | rs4532 | 0.949 | 0.715 | 1.259 | 0.715 |
| *SLC6A3* | rs4975646 | 0.840 | 0.614 | 1.149 | 0.275 |
| *DRD4* | rs936461 | 1.316 | 0.995 | 1.741 | 0.054 |
| *DRD3* | rs963468 | 0.939 | 0.710 | 1.241 | 0.657 |
| *DRD3* | rs167771 | 0.799 | 0.544 | 1.171 | 0.250 |
| *DRD2* | rs2283265 | 0.801 | 0.568 | 1.129 | 0.205 |
| *SLC6A3* | rs2617605 | 0.919 | 0.690 | 1.225 | 0.566 |
| *DRD2* | rs2734842 | 0.915 | 0.691 | 1.212 | 0.535 |
| *DRD2/ANKK1* | rs2734849 | 1.268 | 0.967 | 1.662 | 0.086 |
| *DRD3* | rs324035 | 0.852 | 0.590 | 1.229 | 0.391 |
| *DRD2* | rs6277 | 1.276 | 0.973 | 1.673 | 0.078 |
| *DRD2* | rs6279 | 0.899 | 0.676 | 1.196 | 0.465 |
| *DRD4* | rs11246226 | 0.998 | 0.764 | 1.302 | 0.986 |
| *DRD3* | rs167770 | 1.017 | 0.727 | 1.422 | 0.921 |
| *SLC6A3* | rs464049 | 1.029 | 0.784 | 1.349 | 0.838 |
| *DRD3* | rs7633291 | 1.278 | 0.859 | 1.902 | 0.227 |
| *DRD3* | rs9817063 | 1.250 | 0.939 | 1.664 | 0.126 |
| *SLC6A3* | rs1048953 | 0.901 | 0.669 | 1.213 | 0.493 |
| *DRD2* | rs1076560 | 0.772 | 0.543 | 1.098 | 0.150 |
| *DRD3* | rs1587756 | 1.188 | 0.753 | 1.875 | 0.458 |
| *DRD3* | rs1800828 | 1.183 | 0.814 | 1.718 | 0.378 |
| *SLC6A3* | rs250686 | 1.053 | 0.792 | 1.401 | 0.720 |
| *DRD3* | rs3773678 | 1.164 | 0.755 | 1.794 | 0.491 |
| *DRD3* | rs6280 | 0.953 | 0.688 | 1.319 | 0.771 |
| *SLC6A3* | rs3863145 | 0.876 | 0.623 | 1.231 | 0.446 |

OR – odds ratio;

CI – lower and upper bound 95% confidence intervals;

ORs are reported for the risk of HPRL attributable to the rare allele vs common allele.
