# Supplementary material for: A pharmacogenetic study of patients with schizophrenia from West Siberia gets insight into dopaminergic mechanisms of antipsychotic-induced hyperprolactinemia
Source: BMC Med Genet. 2019 Apr 9;20(Suppl 1):47. doi: 10.1186/s12881-019-0773-3 (PMC6454588; doi:10.1186/s12881-019-0773-3)
Supplement: Supplementary file 2 — Table S2. Genotype and allele frequencies for MAO gene polymorphisms. (DOC 45 kb) [file 12881_2019_773_MOESM2_ESM.doc]

**Supplementary table 2**

Genotype and allele frequencies for *MAO* gene polymorphisms

| Gender | SNP | Genotypes/alleles | Patients with HPRL, % | Patients without HPRL, % |
| --- | --- | --- | --- | --- |
| Females | rs1799836 | A/A | 44 (34.4%) | 23 (24.5%) |
|  |  | G/A | 48 (37.5%) | 51 (54.3%) |
|  |  | G/G | 36 (28.1%) | 20 (21.3%) |
|  |  | A | 68 (53.1%) | 49 (51.6%) |
|  |  | G | 60 (46.9%) | 45 (48.4%) |
|  | rs1137070 | C/C | 64 (50.0%) | 45 (47.4%) |
|  |  | C/T | 52 (40.6%) | 38 (40.0%) |
|  |  | T/T | 12 (9.4%) | 12 (12.6%) |
|  |  | C | 90 (70.3%) | 64 (67.4%) |
|  |  | T | 38 (29.7%) | 31 (32.6%) |
|  | rs6323 | T/T | 63 (49.2%) | 44 (46.3%) |
|  |  | G/T | 53 (41.4%) | 41 (43.2%) |
|  |  | G/G | 12 (9.4%) | 10 (10.5%) |
|  |  | T | 89 (69.9%) | 65 (67.9%) |
|  |  | G | 39 (30.1%) | 30 (32.1%) |
| Males | rs1799836 | A | 63 (66.3%) | 64 (54.2%) |
|  |  | G | 32 (33.7%) | 54 (45.8%) |
|  | rs1137070 | C | 59 (62.8%) | 88 (75.9%) |
|  |  | T | 35 (37.2%) | 28 (24.1%) |
|  | rs6323 | T | 59 (62.1%) | 89 (76.1%) |
|  |  | G | 36 (37.9%) | 28 (23.9%) |
