# Supplementary material for: A pharmacogenetic study of patients with schizophrenia from West Siberia gets insight into dopaminergic mechanisms of antipsychotic-induced hyperprolactinemia
Source: BMC Med Genet. 2019 Apr 9;20(Suppl 1):47. doi: 10.1186/s12881-019-0773-3 (PMC6454588; doi:10.1186/s12881-019-0773-3)
Supplement: Supplementary file 3 — Table S3. Genotype and allele frequencies for all polymorphisms of studied genes in the subgroup of patients using the risperidone/paliperidone. (DOC 275 kb) [file 12881_2019_773_MOESM3_ESM.doc]

**Supplementary table 3**

Genotype and allele frequencies for all polymorphisms of studied genes in the subgroup of patients using the risperidone/paliperidone

| Gene | SNP | Genotypes/alleles | Patients with HPRL, % | Patients without HPRL, % |
| --- | --- | --- | --- | --- |
| *DRD1* | rs4532 | C/C | 5 (9.3 %) | 2 (13.3 %) |
|  |  | C/T | 18 (33.3 %) | 9 (60.0 %) |
|  |  | T/T | 31 (57.4 %) | 4 (26.7 %) |
|  |  | C | 14 (25.9 %) | 6 (43.3 %) |
|  |  | T | 40 (74.1 %) | 9 (56.7 %) |
| *DRD2* | rs6275 | T/T | 9 (21.4 %) | 3 (42.9 %) |
|  |  | T/C | 14 (33.3 %) | 3 (42.9 %) |
|  |  | C/C | 19 (45.2 %) | 1 (14.3 %) |
|  |  | T | 16 (38.1 %) | 5 (64.3 %) |
|  |  | C | 26 (61.9 %) | 2 (35.7 %) |
| *DRD2* | rs6277 | C/C | 12 (22.2 %) | 8 (57.1 %) |
|  |  | C/T | 26 (48.1 %) | 3 (21.4 %) |
|  |  | T/T | 16 (29.6 %) | 3 (21.4 %) |
|  |  | C | 25 (46.3 %) | 10 (67.9 %) |
|  |  | T | 29 (53.7 %) | 4 (32.1 %) |
| *DRD2* | rs1076560 | C/C | 36 (72.0 %) | 10 (66.7 %) |
|  |  | C/A | 13 (26.0 %) | 4 (26.7 %) |
|  |  | A/A | 1 (2.0 %) | 1 (6.7 %) |
|  |  | C | 43 (85.0 %) | 12 (80.0 %) |
|  |  | A | 7 (15.0 %) | 3 (20.0 %) |
| *DRD2* | rs1801028 | C/C | 48 (90.6 %) | 15 (100.0 %) |
|  |  | C/G | 5 (9.4 %) | 0 |
|  |  | G/G | 0 | 0 |
|  |  | C | 51 (95.3 %) | 15 (100.0 %) |
|  |  | G | 2 (4.7 %) | 0 |
| *DRD2* | rs4245147 | C/C | 10 (18.5 %) | 4 (26.7 %) |
|  |  | C/T | 27 (50.0 %) | 5 (33.3 %) |
|  |  | T/T | 17 (31.5 %) | 6 (40.0 %) |
|  |  | C | 23 (43.5 %) | 6 (43.3 %) |
|  |  | T | 31 (56.5 %) | 9 (56.7 %) |
| *DRD2* | rs2283265 | G/G | 38 (70.4 %) | 9 (64.3 %) |
|  |  | G/T | 15 (27.8 %) | 4 (28.6 %) |
|  |  | T/T | 1 (1.9 %) | 1 (7.1 %) |
|  |  | G | 46 (84.3 %) | 11 (78.6 %) |
|  |  | T | 8 (15.7 %) | 3 (21.4 %) |
| *DRD2* | rs6279 | G/G | 27 (50.0 %) | 4 (28.6 %) |
|  |  | G/C | 21 (38.9 %) | 7 (50.0 %) |
|  |  | C/C | 6 (11.1 %) | 3 (21.4 %) |
|  |  | G | 37 (69.4 %) | 8 (53.6 %) |
|  |  | C | 17 (30.6 %) | 6 (46.4 %) |
| *DRD2* | rs1076562 | G/G | 33 (61.1 %) | 6 (40.0 %) |
|  |  | G/A | 16 (29.6 %) | 7 (46.7 %) |
|  |  | A/A | 5 (9.3 %) | 2 (13.3 %) |
|  |  | G | 41 (75.9 %) | 10 (63.3 %) |
|  |  | A | 13 (24.1 %) | 5 (36.7 %) |
| *DRD2* | rs2734842 | C/C | 7 (13.0 %) | 3 (21.4 %) |
|  |  | C/G | 20 (37.0 %) | 7 (50.0 %) |
|  |  | G/G | 27 (50.0 %) | 4 (28.6 %) |
|  |  | C | 17 (31.5 %) | 6 (46.4 %) |
|  |  | G | 37 (68.5 %) | 8 (53.6 %) |
| *DRD2/*  *ANKK1* | rs2734849 | C/C | 14 (25.9 %) | 3 (21.4 %) |
|  |  | C/T | 26 (48.1 %) | 3 (21.4 %) |
|  |  | T/T | 14 (25.9 %) | 8 (57.1 %) |
|  |  | C | 27 (50.0 %) | 4 (32.1 %) |
|  |  | T | 27 (50.0 %) | 10 (67.9 %) |
| *DRD3* | rs11721264 | A/A | 4 (7.4 %) | 1 (6.7 %) |
|  |  | A/G | 19 (35.2 %) | 2 (13.3 %) |
|  |  | G/G | 31 (57.4 %) | 12 (80.0%) |
|  |  | A | 14 (25.0 %) | 2 (13.3 %) |
|  |  | G | 40 (75.0 %) | 13 (86.7 %) |
| *DRD3* | rs167770 | G/G | 4 (7.4 %) | 1 (6.7 %) |
|  |  | G/A | 19 (35.2 %) | 2 (13.3 %) |
|  |  | A/A | 31 (57.4 %) | 12 (80.0 %) |
|  |  | G | 14 (25.0 %) | 2 (13.3 %) |
|  |  | A | 40 (75.0 %) | 13 (86.7 %) |
| *DRD3* | rs3773678 | C/C | 38 (76.0 %) | 13 (86.7 %) |
|  |  | C/T | 12 (24.0 %) | 2 (13.3 %) |
|  |  | T/T | 0 | 0 |
|  |  | C | 44 (88.0 %) | 14 (93.3 %) |
|  |  | T | 6 (12.0 %) | 1 (6.7 %) |
| *DRD3* | rs963468 | A/A | 9 (16.7 %) | 5 (33.3 %) |
|  |  | A/G | 31 (57.4 %) | 8 (53.3 %) |
|  |  | G/G | 14 (25.9 %) | 2 (13.3 %) |
|  |  | A | 25 (45.4 %) | 9 (60.0 %) |
|  |  | G | 29 (54.6 %) | 6 (40.0 %) |
| *DRD3* | rs7633291 | G/G | 2 (3.7 %) | 0 |
|  |  | G/T | 17 (31.5 %) | 3 (20.0 %) |
|  |  | T/T | 35 (64.8 %) | 12 (80.0 %) |
|  |  | G | 10 (19.4 %) | 2 (10.0 %) |
|  |  | T | 44 (80.6 %) | 13 (90.0 %) |
| *DRD3* | rs2134655 | G/G | 27 (50.0 %) | 8 (53.3 %) |
|  |  | G/A | 26 (48.1 %) | 6 (40.0 %) |
|  |  | A/A | 1 (1.9 %) | 1 (6.7 %) |
|  |  | G | 40 (74.1 %) | 11 (73.3 %) |
|  |  | A | 14 (25.9 %) | 4 (26.7 %) |
| *DRD3* | rs9817063 | C/C | 13 (24.1 %) | 6 (40.0 %) |
|  |  | C/T | 30 (55.6 %) | 7 (46.7 %) |
|  |  | T/T | 11 (20.4 %) | 2 (13.3 %) |
|  |  | C | 28 (51.9 %) | 9 (63.3 %) |
|  |  | T | 26 (48.1 %) | 6 (36.7 %) |
| *DRD3* | rs324035 | C/C | 37 (68.5 %) | 12 (85.7 %) |
|  |  | C/A | 17 (31.5 %) | 2 (14.3 %) |
|  |  | A/A | 0 | 0 |
|  |  | C | 46 (84.3 %) | 13 (92.9 %) |
|  |  | A | 8 (15.7 %) | 1 (7.1 %) |
| *DRD3* | rs1800828 | C/C | 2 (4.0 %) | 0 |
|  |  | C/G | 20 (40.0 %) | 3 (20.0 %) |
|  |  | G/G | 28 (56.0 %) | 12 (80.0 %) |
|  |  | C | 12 (24.0 %) | 2 (10.0 %) |
|  |  | G | 38 (76.0 %) | 13 (90.0 %) |
| *DRD3* | rs167771 | A/A | 37 (69.8 %) | 10 (83.3 %) |
|  |  | G/A | 16 (30.2 %) | 2 (16.7 %) |
|  |  | G/G | 0 | 0 |
|  |  | A | 45 (84.9 %) | 11 (91.7 %) |
|  |  | G | 8 (15.1 %) | 1 (8.3 %) |
| *DRD3* | rs6280 | C/C | 3 (6.0 %) | 1 (6.7 %) |
|  |  | C/T | 22 (44.0 %) | 2 (13.3 %) |
|  |  | T/T | 25 (50.0 %) | 12 (80.0 %) |
|  |  | C | 14 (28.0 %) | 2 (13.3 %) |
|  |  | T | 36 (72.0 %) | 13 (86.7 %) |
| *DRD3* | rs1587756 | T/T | 37 (74.0 %) | 13 (86.7 %) |
|  |  | T/C | 12 (24.0 %) | 2 (13.3 %) |
|  |  | C/C | 1 (2.0 %) | 0 |
|  |  | T | 43 (86.0 %) | 14 (93.3 %) |
|  |  | C | 7 (14.0 %) | 1 (6.7 %) |
| *DRD4* | rs3758653 | T/T | 39 (72.2 %) | 11 (73.3 %) |
|  |  | T/C | 13 (24.1 %) | 4 (26.7 %) |
|  |  | C/C | 2 (3.7 %) | 0 |
|  |  | T | 46 (84.3 %) | 13 (86.7 %) |
|  |  | C | 8 (15.7 %) | 2 (13.3 %) |
| *DRD4* | rs11246226 | C/C | 9 (16.7 %) | 2 (13.3 %) |
|  |  | C/A | 31 (57.4 %) | 5 (33.3 %) |
|  |  | A/A | 14 (25.9 %) | 8 (53.3 %) |
|  |  | C | 25 (45.4 %) | 5 (30.0 %) |
|  |  | A | 29 (54.6 %) | 10 (70.0 %) |
| *DRD4* | rs936461 | G/G | 19 (37.3 %) | 7 (50.0 %) |
|  |  | G/A | 26 (51.0 %) | 6 (42.9 %) |
|  |  | A/A | 6 (11.7 %) | 1 (7.1 %) |
|  |  | G | 32 (62.7 %) | 10 (71.4 %) |
|  |  | A | 19 (37.3 %) | 4 (28.6 %) |
| *SLC6A3* | rs3756450 | C/C | 3 (5.6 %) | 1 (6.7%) |
|  |  | C/T | 14 (25.9 %) | 4 (26.7 %) |
|  |  | T/T | 37 (68.5 %) | 10 (66.7 %) |
|  |  | C | 10 (18.5 %) | 3 (20.0 %) |
|  |  | T | 44 (81.5 %) | 12 (81.5 %) |
| *SLC6A3* | rs2550956 | T/T | 6 (11.1 %) | 2 (13.3 %) |
|  |  | T/C | 29 (53.7 %) | 11 (73.3 %) |
|  |  | C/C | 19 (35.2 %) | 2 (13.3 %) |
|  |  | T | 21 (38.0 %) | 7.5 (50.0 %) |
|  |  | C | 33 (62.0 %) | 7.5 (50.0 %) |
| *SLC6A3* | rs6347 | A/A | 37 (68.5 %) | 10 (66.7 %) |
|  |  | A/G | 12 (22.2 %) | 3 (20.0 %) |
|  |  | G/G | 5 (9.3 %) | 2 (13.3 %) |
|  |  | A | 43 (79.6 %) | 12 (76.7 %) |
|  |  | G | 11 (20.4 %) | 3 (23.3 %) |
| *SLC6A3* | rs2617605 | G/G | 7 (13.0 %) | 1 (7.1 %) |
|  |  | G/A | 25 (46.3 %) | 7 (50.0 %) |
|  |  | A/A | 22 (40.7 %) | 6 (42.9 %) |
|  |  | G | 19 (36.1 %) | 4 (32.1 %) |
|  |  | A | 35 (63.9 %) | 10 (67.9 %) |
| *SLC6A3* | rs3863145 | T/T | 3 (5.2 %) | 1 (5.9 %) |
|  |  | T/C | 14 (24.1 %) | 8 (47.1 %) |
|  |  | C/C | 41 (70.7 %) | 8 (47.1 %) |
|  |  | T | 10 (17.2 %) | 5 (29.4 %) |
|  |  | C | 48 (82.8 %) | 12 (70.6 %) |
| *SLC6A3* | rs250686 | A/A | 10 (20.0 %) | 4 (26.7 %) |
|  |  | A/G | 25 (50.0 %) | 9 (60.0 %) |
|  |  | G/G | 15 (30.0 %) | 2 (13.3 %) |
|  |  | A | 23 (45.0 %) | 9 (56.7 %) |
|  |  | G | 27 (55.0 %) | 6 (43.3 %) |
| *SLC6A3* | rs464049 | C/C | 15 (27.8 %) | 2 (13.3 %) |
|  |  | C/T | 28 (51.9 %) | 9 (60.0 %) |
|  |  | T/T | 11 (20.4 %) | 4 (26.7 %) |
|  |  | C | 29 (53.7 %) | 6 (43.3 %) |
|  |  | T | 15 (46.3 %) | 9 (56.7 %) |
| *SLC6A3* | rs4975646 | G/G | 32 (59.3 %) | 7 (46.7 %) |
|  |  | G/A | 19 (35.2 %) | 6 (40.0 %) |
|  |  | A/A | 3 (5.6 %) | 2 (13.3 %) |
|  |  | G | 42 (76.9 %) | 10 (66.7 %) |
|  |  | A | 12 (23.1 %) | 5 (33.3 %) |
| *SLC6A3* | rs1048953 | T/T | 5 (10.0 %) | 3 (20.0 %) |
|  |  | T/C | 22 (44.0 %) | 5 (33.3 %) |
|  |  | C/C | 23 (46.0 %) | 7 (46.7 %) |
|  |  | T | 16 (32.0 %) | 6 (36.7 %) |
|  |  | C | 34 (68.0 %) | 9 (63.3 %) |
| *SLC6A3* | rs11133767 | G/G | 8 (14.8 %) | 2 (13.3 %) |
|  |  | G/A | 21 (38.9 %) | 1 (6.7 %) |
|  |  | A/A | 25 (46.3 %) | 12 (80.0 %) |
|  |  | G | 19 (34.3 %) | 3 (16.7 %) |
|  |  | A | 35 (65.7 %) | 12 (83.3 %) |
| *SLC6A3* | rs27048 | C/C | 16 (29.6 %) | 7 (46.7 %) |
|  |  | C/T | 23 (42.6 %) | 6 (40.0 %) |
|  |  | T/T | 15 (27.8 %) | 2 (13.3 %) |
|  |  | C | 28 (50.9 %) | 10 (66.7 %) |
|  |  | T | 26 (49.1 %) | 5 (33.3 %) |
| *SLC6A3* | rs40184 | G/G | 23 (39.0 %) | 3 (17.6 %) |
|  |  | G/A | 25 (42.4 %) | 8 (47.1 %) |
|  |  | A/A | 11 (18.6 %) | 6 (35.3 %) |
|  |  | G | 36 (60.2 %) | 7 (41.2 %) |
|  |  | A | 23 (39.8 %) | 10 (58.8 %) |
