# Supplementary material for: A pharmacogenetic study of patients with schizophrenia from West Siberia gets insight into dopaminergic mechanisms of antipsychotic-induced hyperprolactinemia
Source: BMC Med Genet. 2019 Apr 9;20(Suppl 1):47. doi: 10.1186/s12881-019-0773-3 (PMC6454588; doi:10.1186/s12881-019-0773-3)
Supplement: Supplementary file 5 — Table S5. Analysis of association between HPRL and polymorphisms in MAO genes for female/male patients in risperidone/paliperidone group. (DOC 34 kb) [file 12881_2019_773_MOESM5_ESM.doc]

**Supplementary table 5**

Analysis of association between HPRL and polymorphisms in *MAO* genes for female/male patients in risperidone/paliperidone group

| Gender | SNP | OR | 95% CI  Lower bound | 95% CI  Upper bound | *p*-value |
| --- | --- | --- | --- | --- | --- |
| Females | rs1799836 | 2.873 | 0.679 | 12.155 | 0.152 |
|  | rs1137070 | 1.154 | 0.225 | 5.914 | 0.863 |
|  | rs6323 | 1.154 | 0.225 | 5.914 | 0.863 |
| Males | rs1799836 | 0.725 | 0.287 | 1.834 | 0.497 |
|  | rs1137070 | 0.990 | 0.366 | 2.678 | 0.985 |
|  | rs6323 | 0.990 | 0.366 | 2.678 | 0.985 |

OR – odds ratio;

CI – lower and upper bound 95% confidence intervals;

ORs are reported for the risk of HPRL attributable to the rare allele vs common allele.
